# Evaluation of Bedtime vs. Morning Levothyroxine Intake to Control Hypothyroidism in Older Patients: A Pragmatic Crossover Randomized Clinical Trial

**DOI:** 10.3389/fmed.2022.828762

**Published:** 2022-06-23

**Authors:** Renato Bandeira de Mello, Karina Giassi, Gabriela Stahl, Maria Luisa Machado Assis, Marina Siqueira Flores, Bruna Cambrussi de Lima, Vanessa Piccoli, Ticiana da Costa Rodrigues

**Affiliations:** ^1^Post-graduate Program in Health Sciences: Endocrinology, Universidade Federal do Rio Grande do Sul, Porto Alegre, Brazil; ^2^School of Medicine, Universidade Federal do Rio Grande do Sul, Porto Alegre, Brazil; ^3^Geriatric Unit/Internal Medicine Division, Hospital de Clínicas de Porto Alegre, Porto Alegre, Brazil; ^4^Endocrinology Division, Hospital de Clínicas de Porto Alegre, Porto Alegre, Brazil

**Keywords:** older adults, thyroxine, hypothyroidism, clinical trial, treatment, evening

## Abstract

**Introduction:**

Drug scheduling in older adults can be a challenge, especially considering polypharmacy, physical dependency, and possible drug interactions. Properly testing alternative treatment regimens could therefore help to overcome treatment barriers. Hypothyroidism is a prevalent condition in older adults, however, studies evaluating L-thyroxine treatment effectiveness in this specific age group are still lacking. Most studies testing an evening administration of levothyroxine were mainly composed of younger adults. Therefore, this trial is aimed to assess if evening levothyroxine (LT4) administration can effectively control hypothyroidism in older patients.

**Materials and Methods:**

A randomized crossover clinical trial was conducted between June 2018 and March 2020 at the Hospital de Clínicas de Porto Alegre, a teaching hospital in Brazil, to compare the efficacy of morning and evening administration of LT4 for hypothyroidism control in older patients. The study protocol is published elsewhere. A total of 201 participants, ≥60 years old, with primary hypothyroidism treated with LT4 for at least 6 months and on stable doses for at least 3 months were included. Participants were randomly assigned to a starting group of morning LT4 intake (60 min before breakfast) or bedtime LT4 intake (60 min after the last meal). After ≥12 weeks of follow-up, a crossover between strategies was performed. The primary outcome was the change in serum thyrotropin (Thyroid-Stimulating Hormone; TSH) levels after 12 weeks of each LT4 administration regimen.

**Results:**

A total of 201 participants with mean age of 72.4 ± 7.2 years were included, out of which 84.1% were women; baseline characteristics and frequency of controlled hypothyroidism were similar between groups. Mean baseline TSH was 3.43 ± 0.25 mUI/L. In total, 118 participants attended three meetings, allowing 135 comparisons by crossover analytic strategy. Mean TSH levels after follow-up were 2.95 ± 2.86 in the morning group and 3.64 ± 2.86 in the bedtime group, *p* = 0.107.

**Discussion:**

Thyroid-Stimulating Hormone levels and frequency of controlled hypothyroidism were similar during the follow-up period regardless of the treatment regimen (morning or bedtime).

## Introduction

The reduced production of thyroid hormones results in a clinical state named hypothyroidism; the thyroid gland (primary) is affected in 99% of the cases and autoimmunity is the major etiology (90% of the cases) in sufficient iodine areas (1). Robust epidemiologic data on population thyroid function levels come from large studies from Europe and the United States (2–4). The mean annual incidence of spontaneous hypothyroidism in the Whickham Survey Cohort (1972–1993) was 35 cases per 10,000 women and 6 cases per 10,000 men (3). The National Health and Nutrition Examination Survey–NHANES–(1988–1994) reported a 4.6% prevalence of hypothyroidism (0.3% clinical hypothyroidism and 4.3% subclinical hypothyroidism) and a 97.5% increase in Thyroid-Stimulating Hormone (TSH) levels with aging (4). Hypothyroidism treatment seeks to resolve the signals and symptoms of the condition and to re-establish serum TSH levels to reference limits (5).

When thyroxine sodium salt was first introduced in 1949, levothyroxine (LT4) became a treatment option for hypothyroidism and, eventually, monotherapy was established as the standard choice of therapy (6). Tablets need the physiologic acid gastric environment to dissolve, and the small intestine (duodenum and jejuno-ileum) is the main location of absorption (7, 8). Healthy volunteers can reach 60–80% of levothyroxine bioavailability and require about 2–3 h from the oral intake to reach maximum serum concentration (9–11). Changes in pharmacokinetics can impair levothyroxine replacement and are caused by (1) gastrointestinal disorders, (2) drug-food interactions, (3) drug-drug interactions, and (4) old age (12, 13). In a study with euthyroid participants over 70 years old, thyroxine absorption was 9.4% lower (62.8 ± 13.5% SD vs. 69.3 ± 11.9% SD; *p* < 0.001) than for younger counterparts (13). A complex drug regimen is prevalent in older ages and can result in negative clinical effects, such as lower adherence (14). In cross-sectional surveys, the non-compliance of levothyroxine, defined as proportion of days covered (PDC) as <80%, reaches 50% or more of individuals with hypothyroid (15, 16) and is associated with the presence of other comorbidities (17). Furthermore, a Brazilian study showed that 40.6% of the samples used levothyroxine together with other medication, such as proton-pump inhibitors (PPIs) and calcium and iron supplements (18).

Other levothyroxine intake regimens have thus been proposed to improve treatment effectiveness. As an example, Bevenga et al. (19) showed that preponing levothyroxine intake from 15–20 to 60 min before breakfast resulted in higher TSH suppression during a follow-up of 4–15 months. In a crossover study based on their pilot trial (20), Bolk et al. assessed the effects of bedtime levothyroxine administration vs. administration 30 min before breakfast. At the end of 24 weeks, LT4 bedtime intake was as effective as morning intake regarding thyroid hormone levels (mean age 48 years) (21). Recently, a meta-analysis that grouped six studies (*n* = 527) on LT4 timing; altogether, LT4 administration for 30 min between the administration and before breakfast and at bedtime had no statistically significant differences in TSH levels [standard mean differences (SEM) = −0.19, 95% CI: −0.53, 0.15; *p* = 0.28] (22).

Drug scheduling in older adults can be a challenge, especially considering polypharmacy, dependency, and possible pharmacological interactions. Proper tests of alternative drug regimens can thus help to overcome treatment barriers. Hypothyroidism is a prevalent condition in older adults, however, studies evaluating L-thyroxine treatment effectiveness in this specific age group are still lacking. Most studies, which tested a bedtime administration of levothyroxine, were mainly composed of younger adults (19–21). Therefore, this trial seeks to assess if bedtime levothyroxine (LT4) administration can effectively control hypothyroidism in older patients.

## Materials and Methods

The detailed trial protocol was published elsewhere (23). This research was approved by the Hospital de Clínicas de Porto Alegre Research Ethics Committee, in accordance with the principles of the Declaration of Helsinki and Good Clinical Practice guidelines (24). All participants provided written informed consent. *The Fundo de Incentivo à Pesquisa e Eventos* (FIPE) from the Hospital de Clínicas provided primary financial support for the trial. The funder had no role in the design, analysis, or reporting of the trial. National Clinical Trial Identifier number is NCT03614988.

## Study Design and Setting

Study design and setting: Pragmatic, randomized, crossover clinical trial was conducted at the Endocrinology and Internal Medicine Outpatient clinics at the Hospital de Clínicas de Porto Alegre, Brazil.

### Eligibility Criteria

Participants were identified from the Endocrinology and Internal Medicine Clinics at the Hospital de Clínicas de Porto Alegre, a teaching hospital from Universidade Federal do Rio Grande do Sul, a top-ranked university in Brazil. Inclusion criteria were outpatients ≥60 years old with primary hypothyroidism who had been using LT4 for at least 6 months and were on stable doses for the last 3 months. Exclusion criteria were severe chronic diseases, such as end-stage renal failure, severe chronic obstructive pulmonary disease (COPD), severe hepatic failure, advanced cancer, dementia, thyroid cancer, heart failure (functional class IV), patients under palliative care, three or more hospital admissions during the last year due to heart failure decompensation, and refusal to participate.

## Randomization

A randomization list stratified by sex and age (≥75 years) was created using a web-based program (http://www.randomization.com/). Interchangeable random blocks/variables' blocked randomization strategy was chosen to conceal allocation and to avoid intergroup disparities from the predicted sample size. An independent researcher was responsible for the randomization list and for treatment allocation.

After enrollment, participants underwent randomization in a 1:1 ratio to define the starting treatment regimen.

### Intervention

According to random treatment allocation, participants were instructed to take LT4 tablets 60 min before breakfast (morning strategy) or at bedtime, at least 60 min after the last meal (bedtime strategy). Different from other western countries, Brazilian breakfast is a light meal, being lunch the heaviest meal in a common day. In Brazil, the last meal is usually a light meal like breakfast, mainly based on fruits, bread, milk, and dairy, occurring 2 h after supper (similar but usually lighter than lunch). A crossover was planned to be implemented 12 weeks after the baseline assessment. Since the hypothyroidism-diagnosed patients were already under treatment, the study did not provide LT4 tablets, to encourage them to keep the same drug brand and doses they were used to take before enrollment. The intervention was the drug scheduling itself. Furthermore, levothyroxine intake was pragmatically instructed as in a doctor's office visit, reinforcing it should be taken at least 1 h before breakfast and at least 1 h after the last meal at bedtime. Similarly, as in a usual clinical visit, participants were instructed to take LT4 with water and to avoid taking other medications for at least 60 min. No treatment dose adjustments were conducted by researchers; however, those participants presenting altered TSH levels were instructed to seek their doctors to evaluate the need for LT4 treatment adjustments.

### Blinding

Considering the pragmatic intervention strategy, researchers and participants were not blinded. The researcher responsible for statistical analysis was blinded to the treatment allocation. Moreover, the primary outcome was an objective laboratory test result, not influenced by external perceptions.

## Study Procedures

**Figure 1:**
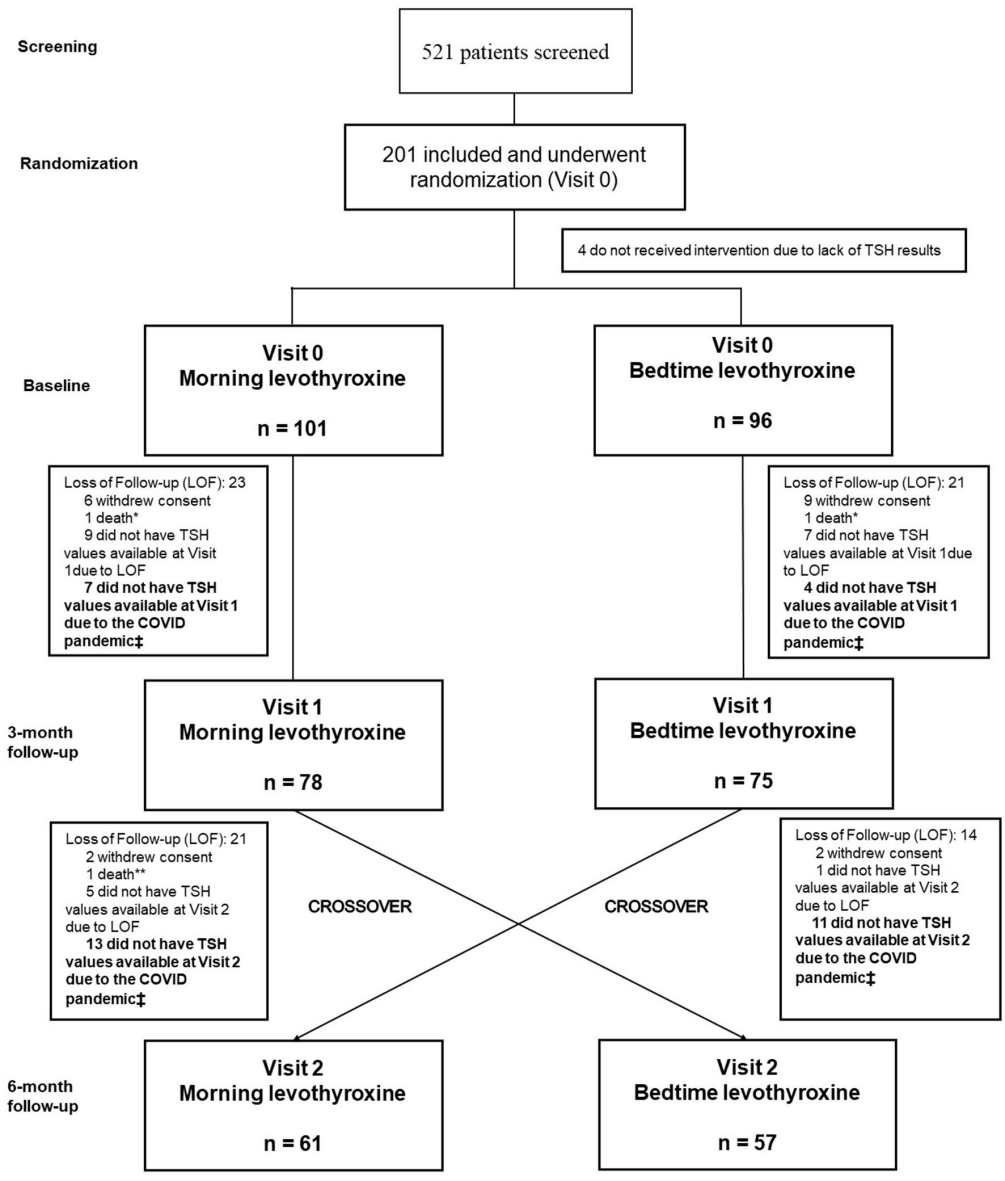
Study design and procedures.

Follow-up, crossover, and study procedures: Randomized participants had to attend three study visits, baseline assessment, and 3- and 6-month follow-up visits. Detailed study procedures for each visit were described elsewhere (23). In short, the first follow-up visit was scheduled for 3 months (12 weeks) after baseline assessment and randomization to the starting treatment regimen to assess thyroid function tests and check for treatment adherence and adverse events during follow-up (Figure 1). At the end of the 3-month follow-up visits, participants were oriented to switch to the alternative treatment regimen and a 6-month follow-up visit was scheduled to reassess thyroid function tests. Although the LT4 has a late peak effect and long half-life, the late carryover effect was not expected since the main outcome would be assessed 12 weeks after the treatment strategy switch. Thus, a wash-out period between strategies was not applied.

During all follow-up visits, independent and dependent variables were collected using a standardized questionnaire: food-LT4 intervals (≤29 mins, 30–59 min, or ≥60 min); drug-LT4 interactions (use of PPIs and calcium, multivitamin, and/or iron supplements within 60 min of levothyroxine intake); and the Barthel Scale, which assesses ten items of activities of daily living (ADLs), ranging from 0 points (fully dependent) to 100 points (fully independent). Adverse events were assessed and recorded. Thyroid function (serum TSH and free T4 levels) was measured by electrochemiluminescence assay; concentrations between 0.27–4.2 mUI/L and 0.93–1.7 ng/dl were considered the normal laboratory range.

The primary outcome was the change in serum TSH levels after 12 and 24 weeks of follow-up from the baseline. Secondary outcomes included the identification of concomitant drugs interfering in LT4 absorption and the evaluation of levothyroxine effectiveness between the two treatment strategies (frequency of hypothyroidism control).

SAS Studio 3.7 was used to calculate the sample size in accordance with the parameters described by Bolk et al. (21). A total of 92 analytic pairs should be included (46 individuals randomized for each starting treatment strategy) to detect a difference of 1.0 mUI/L in mean TSH levels between treatment strategies, considering a standard deviation (SD) between 2.5 and 3.0, with 80% power and 5% significance level. Adding 10% for possible losses and refusals, the sample size should be 100 (50 per starting treatment regimen).

### Statistical Analysis

Quantitative variables with normal distribution were described as mean and SD and variables with non-normal distribution as median and interquartile range (IQR). Qualitative variables were described as absolute and relative frequencies. The treatment effect and carryover effect were analyzed by generalized estimating equation (GEE) models for crossover studies with gamma distribution. A sensitive analysis using General Linear Model (GLM) for repeated measures and a *t*-test for independent samples to compare visit 1 results was also performed and presented in the Supplementary Material section.

Because of the COVID pandemic, this study follow-up was initially restricted in February 2020 and then definitively interrupted at the end of March 2020. Therefore, researchers were unable to collect clinical or laboratory data for 35 enrolled participants, with 11 losses in the 3-month follow-up visit and 24 others in the 6-month follow-up visit.

## Results

### Trial Population

**Figure 2:**
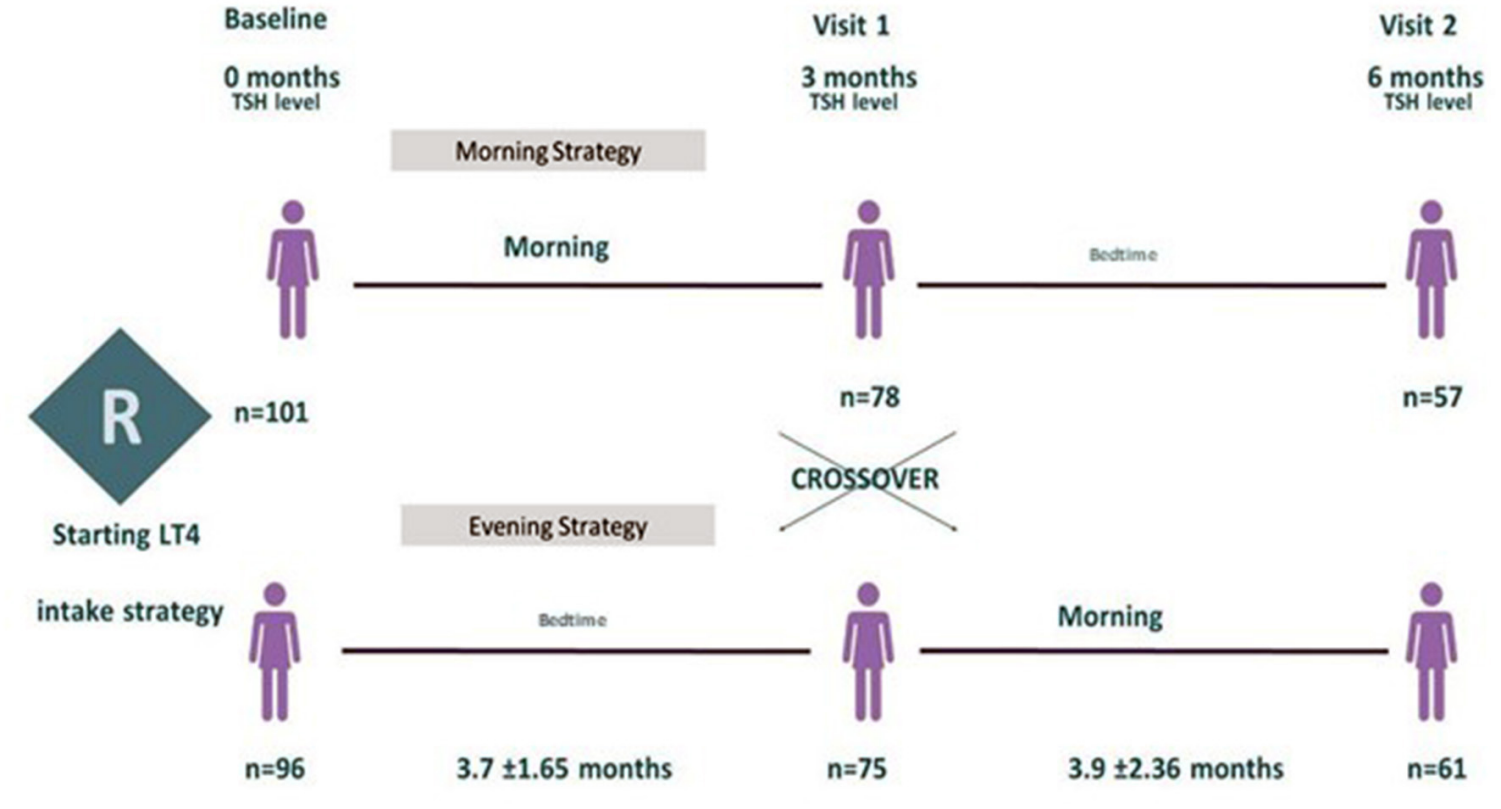
Flowchart of the study displays participants enrollment from screening to study completion. *Unknow causes **Lung Cancer ‡ COVID pandemic imposed research limitations (The Clinical Research Center was closed for non-urgent or not COVID-related researches in February 2020).

We screened 521 outpatients diagnosed with primary hypothyroidism who were at least 60 years old. Of 201 participants who fulfilled the eligibility criteria and underwent randomization, 101 participants were assigned to the morning strategy as the first treatment regimen and 96 to the bedtime strategy. Four patients did not collect thyroid function at baseline and were excluded just after randomization. During follow-up, 135 participants (78 initially assigned to the morning group and 75 assigned to the bedtime group) were evaluated and had blood samples collected for thyroid function tests at visit 1, whereas 118 participants (61 mornings and 57 bedtimes) did so at visit 2 Figure 2 shows the study's inclusion flowchart with losses and enrollment details, restrictions imposed by the COVID pandemic, and follow-up losses.

Table 1 shows the baseline characteristics of the participants. Since the study is a crossover trial, patients have also their own control group; thus, baseline data are summarized and shown as a single group. The mean age was 72.5 ± 7.2 years, 169 participants were women (84.1%), and the main cause of primary hypothyroidism was Hashimoto's thyroiditis (170; 84.6%). The overall median baseline TSH level was 2.59 (IQR 1.28–4.3) mUI/L. Most individuals were taking generic levothyroxine (110; 54.7%) in the morning (199; 99%) and 30–59 min before breakfast (72; 35.8%). The mean levothyroxine dosage was 91.4 ± 45.7 mcg. LT4 doses were stable for a median time of 22.5 months (IQR, 8.2–53.7) before inclusion. Despite multimorbidity, the mean number of concomitant diagnoses was 6 ± 1.9 and participants were mostly functionally independent (Barthel Scale 94 ± 12.1 points). Sensitivity analysis comparing independent groups defined by randomization and direct comparisons at each time-point is presented in a separate document (Supplementary Material). Baseline comparisons between groups are presented in Supplementary Table S1. Supplementary Table S2 presents additional data for LT4 dose adjustments.

**Table 1:** Baseline characteristics of the 201 enrolled participants.

	***n* = 201**
**Characteristic**	**mean** **±SD or median (IQR) or** ***n*** **(%)**
Age (years)	72.5 ± 7.2
Female	169 (84.1)
Years of schooling	5.6 ± 4.2
TSH level mUI/L median (IQR) &	2.59 (1.28–4.30)
Free T4 ng/dl median IQR #	1.35 (1.17–1.58)
Hypothyroidism ethology	170 (84.6)
Hashimoto's Thyroiditis	22 (11.0)
Total Thyroidectomy	7 (3.5)
Radioactive Iodine Therapy for Hyperthyroidism	2 (1.0)
Lobectomy due to benign nodule	
Time of hypothyroidism diagnosis in months - median (IQR)	120 (60–216)
Time on Levothyroxine stable doses in months- median (IQR)	22.5 (8.2–53.7)
Levothyroxine dose (mcg)	91.4 ± 45.7
Usual levothyroxine intake regimen prior to the study	
Morning	199 (99)
Bedtime	2 (1.0)
Interval between levothyroxine and food	
≤ 29 minutes	46 (22.9)
30–59 minutes	72 (35.8)
≥60 minutes	83 (41.3)
Possible interfering medications ∤	
Yes	77 (38.3)
PPI	66 (32.8)
Calcium supplement	16 (8)
Multivitamin supplements	2 (1.0)
Iron supplement	3 (1.5)
No	117 (58.2)
Number of comorbidities	6 ± 1.9
Number of medications in use	7.6 ± 3.1
Functional Capacity (Barthel Scale 0–100 pts)	94 ± 12.1
Baseline TSH level categories on hypothyroidism control status	
TSH <0.27	8 (4)
TSH 0.27–4.2	137 (68.2)
TSH > 4.2	52 (25.9)

*∤ 7 missing values; & 4 missing values; # data collected in 128 participants PPI = proton-pump inhibitors*.

### Treatment Compliance During Follow-Up

At visit 1, in the morning group, 6 (7.6%) participants informed that they were taking LT4 < 29 min before breakfast, 24 (30.7%) were taking it 30–59 min before breakfast and 48 (61.5%) participants informed they were taking LT4 at least 60 min before breakfast. In the bedtime group, 43 (57.4%) participants informed usual LT4 intake at least 60 min after the last meal in accordance with the study's protocol; 27 (36%) were taking LT4 30–59 min after the last meal, and 5 (6.6%) were regularly taking LT4 < 30 min after the last meal. No significant differences were found between groups regarding treatment compliance; however, it may reflect a lack of power due to additional grouping to test this hypothesis. At visit 2, numbers were slightly different, favoring better compliance in the bedtime group (not statistically significant). Morning group: 5 (8.2%) participants were taking LT4 < 30 min before breakfast; 18 (29.5%) participants were LT4 taking 30–59 min, and 38 (62.3%) participants were taking the medication 60 min before breakfast. Bedtime group: 3 (5.2%) participants were taking LT4 < 30 min after the last meal, 10 (17.5%) participants were taking it 30–59 min, and 44 (77.2%) participants were taking LT4 60 or more min after the last meal.

### Bedtime vs. Morning LT4 Intake

According to the crossover analysis, the mean TSH levels (mUI/L) were 2.95 ± 2.86 for LT4 intake in the morning and 3.64 ± 2.86 for the bedtime, with no statistical differences between groups and *p* = 0.107 (Table 2). Sensitivity analysis was performed on test results of participants with stable LT4 doses during the study's follow-up period and results remained similar, however, there is a trend that morning LT4 intake has lower TSH levels when compared to the bedtime intake group (Table 2). A stratified comparison considering three different TSH level categories (low TSH level; normal TSH level; and high TSH level) was also performed, showing no significant differences regarding hypothyroidism control rates (Table 3). Further sensitivity analysis showed no significant differences between groups on both TSH and free thyroxine levels when visit 1 data were analyzed individually through an independent group *t*-test analytic strategy or when GLM for repeated measures was conducted to test mean differences between groups during both study's follow-up visits (Supplementary Tables S3, S4).

**Table 2:** Effects of bedtime vs. morning levothyroxine intake in Thyroid-Stimulating Hormone (TSH) levels (crossover model).

**Total Sample**	**Mean (95%CI)**		
	Morning (*n* = 139)	Evening (*n* = 132)	*p*-value
TSH mUI/L	2.95 (2.47 to 3.43)	3.64 (3.16 to 4.12)	0.107
**Sample with stable LT4 doses during follow-up**	**Mean (95%CI**)		
	Morning (*n* = 115)	Evening (*n* = 115)	*p*-value
TSH mUI/L	2.83 (2.37 to 3.29)	3.56 (3.07 to 4.05)	0.062

**Table 3:** Hypothyroidism control status according to LT4 intake regimen.

	**Visit 1**			**Visit 2**		
	**Morning (*n* = 78)**	**Bedtime (*n* = 75)**	*p*-value^*^	**Morning (*n* = 61)**	**Bedtime (*n* = 57)**	*p*-value^*^
TSH <0.27 mUI/L	9 (11.5)	6 (8)	0.84	4 (6.6)	5 (8.8)	0.17
TSH 0.27–4.2 mUI/L	50 (64.1)	51 (68)		35 (57.4)	40 (70.2)	
TSH > 4.2 mUI/L	19 (24.4)	18 (24)		22 (36.1)	12 (21.1)	

* *chi-square test*.

The carryover effect was tested and was not observed (*p* = 0.504). Interestingly, of all participants who completed the study, only 22 (10.9%) said they preferred bedtime LT4 intake over morning intake. The most common adverse event was respiratory tract infection; however, no statistical difference was found between treatment intake strategies.

### Interfering Medications

In total, 77 (38.3%) patients were using possibly interfering medications within 60 min of levothyroxine intake, mostly PPIs (66; 32.8%). Different than initially thought, the mean TSH level is lower during morning LT4 intake than during bedtime intake (*p* = 0.033) as described in Table 4. The mean difference of TSH levels according to interfering medication status is presented in Supplementary Table S5.

**Table 4:** Effects of bedtime vs. morning levothyroxine intake in Thyroid-Stimulating Hormone (TSH) levels of participants using interfering drugs^*^ 60 min within levothyroxine administration (crossover model).

	Mean (95%CI)		
	**Morning (*n* = 57)**	**Bedtime (*n* = 52)**	***p*-value**
TSH mUI/L	2.81 (2.14 to 3.48)	4.10 (2.9 to 5.3)	0.033

* *Proton-pump inhibitors and calcium, multivitamin, and iron supplements*.

## Discussion

This pragmatic crossover clinical trial compared the effectiveness of two different LT4 treatment regimens to control hypothyroidism in older patients already under stable LT4 doses. No significant differences were found between bedtime or morning LT4 intake regarding TSH levels and hyperthyroidism control rates. Results remained similar after a sensitivity analysis that included only participants who were receiving stable doses of LT4 during the study's follow-up. To the authors' knowledge, this is the first study to compare two different LT4 intake regimens in older patients in a pragmatic real-world clinical trial that includes multimorbid participants and those using interfering medications.

This study's results corroborate with previous publications that analyzed younger populations (25–30). Rajput et al. observed two groups (mean age: 34.30 ± 11.82 years) in a parallel clinical trial, i.e., 152 levothyroxine drug-naïve patients. After 12 weeks of levothyroxine treatment, they found no significant differences in mean TSH levels between LT_4_ intake 2 h after dinner and 30 min before breakfast (3.27 mUI/L ± 4.19 vs. 5.13 mUI/L ± 9.36; *p* = 0.31) (25). Ahmed et al. (26) and Srivastava et al. (27) tested a similar strategy but increased the LT4 intake interval to 60 min before breakfast. A total of 82 participants were included and, after 3 months of follow-up, no statistical significance was found in TSH levels between groups. The mean difference in TSH reduction from baseline was 13.6 ± 22.2 mUI/L in the morning group and 11.3 ± 22.5 mUI/L in the bedtime group, *p* = 0.63. Srivastava et al. (27) tested the same hypothesis using a crossover model; however, results were analyzed using *t*-test for independent samples, an analytic approach that can overestimate results in crossover studies, especially if a non-parametric variable is the main study outcome. In total, 59 participants were included and the bedtime LT4 administration group had better outcomes.

Different from other studies on alternative LT4 regimens for hypothyroidism control, this crossover study design intended to bring real-world treatment data. Multimorbid and polypharmacy-exposed older participants were included to provide data on hypothyroidism treatment effectiveness in a pragmatic real-world clinical setting. Multimorbidity and polypharmacy are a major concern in older patients and a great barrier to an optimal levothyroxine treatment regimen, especially because multimorbidity must be addressed during pharmacologic treatment plans. Thus, patients were pragmatically instructed during a visit to the doctor's office, reinforcing that levothyroxine should be taken at least 1 h before breakfast and at least 1 h before the last meal, at bedtime. Older adults are usually excluded from clinical trials mainly because of strict exclusion criteria about interfering medications and multimorbidity. Although this can better demonstrate treatment efficacy, it can also limit effectiveness tests in real-world scenarios. This study situation is suboptimal, but it simulates clinical practice reality in a pragmatic clinical trial, which compares treatment strategies despite interfering factors. Moreover, as mentioned before, randomization and the crossover design were applied to minimize the effects of known and unknown confusion variables over the primary outcome. Crossover trials are efficient since estimated treatment effects are based either wholly or largely on within-subject variance and contrasts between groups, reducing the contribution of the between-subject component and accurately estimating means for a group of patients. However, individualized treatment plans can be considered, especially when a patient is under interfering medications.

A sensitive analysis that included participants under stable doses of LT4 during the study's follow-up corroborated with previously published findings by Appayadin et al. (28) (TSH mean: 2.83 ± 2.51 mUI/L in the morning group and 3.56 ± 2.68 mUI/L in the bedtime group; *p* = 0.062).

Individuals under interfering medications are commonly excluded from clinical trials on levothyroxine efficacy (25–28). However, Skelin et al. (29) and Bach-Huynhm (30) did not exclude participants using known LT4 interfering medications from their trials. Skeling et al. found no significant differences in TSH levels regarding alternative treatment regimens, possibly because of the lack of power from a small sample size. On the other hand, Bach-Huynhm showed that TSH levels at 24 weeks were significantly lower for fasting LT4 administration than for administration two hours after dinner[1.06 mUI/L, 95% confidence interval (CI), 0.6–1.52 vs. 2.19 mUI/L, 95% CI 1.73–2.65; *p* < 0, 001] (30), though both groups achieved TSH levels categorized as controlled hypothyroidism.

Previous studies (31–33) showed worse levothyroxine absorption related to PPIs and multivitamins, calcium, and iron supplements. In this study, patients in the morning group who used the investigated interfering drugs had lower TSH levels, indicating a trend toward morning intake. However, these results must be cautiously interpreted considering measurement bias (interfering medications data were collected as a dichotomous variable; dosing and time of administration were not registered) or analytical imprecision from the small number of participants in this subgroup analysis (57 analytic pairs). Table 4 shows that the sample size decreases from 135 participant analytic pairs to 57 analytic pairs, making results more imprecise and with larger ranges of confidence intervals. Though the *p*-value is significant, a clear superposition of mean CIs indicates a non-significant result, possibly explained by the study's imprecision to test this specific secondary outcome. Even if a difference was accepted, considering 60% of patients were not taking interfering medications, we assumed a minor impact over the primary outcome, especially because there was a minimal mean TSH level difference between groups defined by the use of intervenient medications.

We emphasize that higher bedtime TSH results are a trend in all pre-specified analyses, which represents a valid and real difference, however, it does not represent a clinically relevant difference since mean TSH levels are still inside the expected target laboratory range categorized as controlled hypothyroidism. Moreover, hypothyroidism control rates were similar between groups. The authors therefore concluded that bedtime LT4 administration can be as effective as morning administration to control hyperthyroidism affecting older adults.

Furthermore, baseline data show a significant heterogeneity regarding levothyroxine intake time, a real-world treatment compliance problem that may influence hypothyroidism control rates in clinical practice. The authors believe that this is another good reason to test alternative treatment regimens in a pragmatic clinical trial since delaying breakfast to favor the absorption of a drug is hard, especially for older adults, an age group frequently affected by multimorbidity (e.g., diabetes, hypertension, and heart failure) and polypharmacy (e.g., antihypertensive and hypoglycemic drugs), conditions to which fasting can impose at least a theoretical risk. This scenario was considered during study planning as an important issue to address in a pragmatic designed study regarding hypothyroidism control in a real-world clinical setting, as we did in this study. However, we observed that the frequency of inappropriate treatment compliance patterns did not change throughout the rest of the study despite of instructions of the investigators, being similar between groups. Maybe it represents a common patient behavior that can influence results in both clinical practice and this specific study.

The main possible limitation of this study is the loss of follow-up by restrictions from the COVID pandemic. However, the final crossover comparison sample size (135 analytical pairs) guaranteed enough power to test for differences with a good precision range, as shown by the small-ranged CIs. Moreover, considering the demonstrated difference of 0.69 mUI/L instead of 1.0 mUI/L (as used to calculate sample size) in TSH levels between groups, 105 included individuals would give 80% power to detect a treatment difference in a two-sided 0.05 significance level, with total samples smaller than the 118 individuals who completed follow-up. Although this study was not blinded, the chosen outcome is an objective laboratory result, minimizing possible external interference. Even though treatment interfering factors (such as the use of interfering drugs and LT4 dose adjustments during follow-up) were considered *a priori* during study planning and proper sensitivity analyzes were conducted, residual confounding secondary to these factors can still impact the study's results. Thus, it should be considered as a possible study limitation as well.

Finally, based on this study's findings, levothyroxine administration at the bedtime was as effective as in the morning to control hyperthyroidism in older patients. However, further pragmatic trials with bigger sample sizes are still needed to confirm these findings and to test the clinical impact of interfering medications over LT4 effectiveness on real-world clinical practice settings and, thus, on hypothyroidism control rates.

## Data Availability Statement

The raw data supporting the conclusions of this article will be made available by the authors, without undue reservation.

## Ethics Statement

The studies involving human participants were reviewed and approved by the Hospital de Clínicas de Porto Alegre Ethics Committee. The patients/participants provided their written informed consent to participate in this study.

## Author Contributions

KG and RM: analysis. KG, RM, and TC: manuscript writing. KG: data collection. RM and TC: study design, revision, and final approval of the manuscript. RM, KG, GS, MM, MS, BC, VP, and TC: data collection phase, manuscript writing process, and approved the manuscript of the International Committee of Medical Journal Editors criteria for authorship have been met. All authors contributed to the article and approved the submitted version.

## Funding

This project was funded by the Hospital de Clínicas de Porto Alegre Fundo de Incentivo á Pesquisa e Eventos (FIPE number 2018-0209). FIPE had no role in the study design, the data collection, analysis or interpretation, drafting the manuscript, or the decision to submit it for publication.

## Conflict of Interest

The authors declare that the research was conducted in the absence of any commercial or financial relationships that could be construed as a potential conflict of interest.

## Publisher's Note

All claims expressed in this article are solely those of the authors and do not necessarily represent those of their affiliated organizations, or those of the publisher, the editors and the reviewers. Any product that may be evaluated in this article, or claim that may be made by its manufacturer, is not guaranteed or endorsed by the publisher.
