# Supplementary material for: Evaluation of Bedtime vs. Morning Levothyroxine Intake to Control Hypothyroidism in Older Patients: A Pragmatic Crossover Randomized Clinical Trial
Source: Front Med (Lausanne). 2022 Jun 23;9:828762. doi: 10.3389/fmed.2022.828762 (PMC9261378; doi:10.3389/fmed.2022.828762)
Supplement: Supplementary file 1 [file Data_Sheet_1.pdf]

Supplemental Data

Evaluation of Bedtime versus Morning Levothyroxine Intake to Control Hypothyroidism in Older Patients: a pragmatic crossover randomized clinical trial

Table S1. Baseline Characteristics according to study group allocation. n =201

**Characteristic**	**mean±SD or median (IQR) or n(%)**		
	**Morning LT4**	**Bedtime LT4**	**p-value**
Age (years)	72.7±7.01	72.2±7.4	0.62
Female	83 (81.4)	86 (86.6%)	0.28
Years of schooling	5.8±4.05	5.4±4.3	0.50
TSH level mUI/L median (IQR)*	2.9 (1.37-4.74)	2.19 (1.16-4.03)	0.12
Free T4 ng/dl median (IQR)*	1.34 (1.13-1.61)	1.38 (1.21-1.58)	0.51
Hashimoto's Thyroiditis	87 (86.1)	83 (83.8)	0.27
Time of hypothyroidism diagnosis in months - median (IQR)*	107 (58-216)	120 (69.5-240)	0.32
Time on Levothyroxine stable doses in months- median (IQR)	23 (7.25-53.7)	21.5 (10-52.25)	0.96
Levothyroxine dose (mcg)	88.35±38.7	94.47±51.85	0.34
Morning levothyroxine intake regimen prior to the study	101 (99)	98 (99)	0.98
Interval between levothyroxine and food			0.31
≤29 minutes	22 (21.6)	24 (24.3)	
30-59 minutes	37 (36.3)	35 (35.3)	
≥60 minutes	43 (42.1)	40 (40.4)	
Possible Interfering medications			
Yes	40 (41.2)	37 (38.1)	0.66
PPI	32 (33)	34 (35.1)	0.76
Calcium supplement	10 (10.3)	6 (6.2)	0.29
Multivitamin supplements	2 (2.1)	0	0.56
Iron supplement	2 (2.1)	(1.0)	0.15
Number of comorbidities	5.83±1.97	6.23±1.96	0.15
Number of medications in use	7.34±3.24	7.88±2.94	0.21
Functional Capacity (Barthel Scale 0–100 pts)	94.9±7.99	93.21±15.38	0.33
Baseline TSH level categories on hypothyroidism control status			
TSH<0.27	6 (5.9)	2 (2.1)	
TSH 0.27-4.2	62 (61.4)	75 (78.1)	0.03
TSH>4.2	33 (32.7)	19 (19.8)	

*Mann-Whitney test; PPI = proton-pump inhibitors

Table S2. Dose adjustments during study´s follow-up visits according to first treatment allocation

R	Morning Intake first		Bedtime Intake first	
	Visit 1<br>Morning Intake dose (Mcg)	Visit 2<br>Bedtime Intake dose (Mcg)	Visit 1<br>Morning Intake dose (Mcg)	Visit 2<br>Bedtime Intake dose (Mcg)
1	150	100	200	150
2	150	100	50	25
3	50	25	112	100
4	75	50	50	25
5	88	75	37.5	50
6	75	100	62.5	75
7	50	25	112	125
8	75	88	200	100
9	25	50	175	150
10	125	88	[redacted]	
11	200	300		
12	75	50		

Line numbers represents the number of participants submitted to LT4 dose adjustments during follow-up

Table S3. Independent group t-test analytic strategy to compare TSH and Free T4 levels at each study time-point. Results expressed as Mean + (95%CI)*

	Visit 0 Baseline** n= 201				Visit 1‡ n= 153				Visit 2‡ n= 118			
	Morning	Bedtime	Mean Diff ¥	p-value	Morning	Bedtime	Mean diff¥	p-value	Morning	Bedtime	Mean diff¥	p-value
TSH mUI/L	3.62 (2.92-4.33)	3.23 (2.50-3.97)	0.39 (-0.62 to 1.40)	0.85	2.71 (2.20-3.21)	3.13 (2.43-3.83)	-0.42 (-1.27 to 0.42)	0.33	3.27 (2.33-4.21)	3.75 (2.85-4.64)	-0.47 (-0.80 to 1.76)	0.64
Free T4 mUI/L	1.38 (1.29-1.47)	1.4 (1.33-1.47)	-0.013 (-0.12 to 0.09)	0.81	1.36 (1.28-1.44)	1.32 (1.24-1.4)	0.03 (-0.07 to 0.15)	0.53	1.32 (1.21-1.43)	1.34 (1.24-1.44)	-0.01 (-0.16 to 0.13)	0.84

*normal distribution assumed **groups according to randomization ‡groups according to LT4 intake prior to TSH results ¥ Mean difference; T4 = thyroxine

Table S4. TSH levels mean differences between groups at each study time-point from 57 participants with 3 TSH repeated measures available. Mean (95% CI)*

	Visit 0 Baseline	Visit 1 12 wk	Visit 2 24 wk	Visit 1 – Visit 0 Mean difference	Visit 2 – Visit 1 Mean difference	Visit 2 – Visit 0 Mean difference
Morning intake first	3.52 (2.42-4.63)	2.7 (2.10-3.32)	3.8 (2.90-4.72)	-0.81 (-2.29 to 0.66); p=0.53	1.1 (-0.22 to 2.42); p=0.13	0.29 (-1.48 to 2.06); p=0.99
Bedtime intake first	3.75 (2.65-4.86)	2.99 (2.38-3.60)	3.27 (2.36-4.18)	-0.75 (-2.16 to 0.63); p=0.54	0.28 (-0.91 to 1.47); p=0.48	-0.48 (-1.97 to 1.00); p=0.54

*General linear model for repeated measures

Table S5. TSH levels mean differences according to interfering medication status. Mean (95% CI)*

Interfering medication	Yes	No	Mean difference	p-value
Visit 0	3.69 (2.74-4.64)	3.2 (2.61-3.79)	- 0.46 (-0.60 to 1.53)	0.38
Visit 1	3.26 (2.49-4.03)	2.68 (2.19-3.17)	-0.57 (-1.45 to 0.3)	0.19
Visit 2	3.2 (2.34-4.06)	3.7 (2.78-4,62.)	0.5 (-1.8 to 0.82)	0.45

*independent t-test
